# Dr. Royal E. Cochrane

**Published:** 1910-12

**Authors:** 


### OBITUARY

---

Dr. Royal E. Cochrane of Penfield, N. Y., died of angina pectoris at his home in that village, October 22, 1910, aged 66 years. He was a Civil War veteran and a medical graduate of the University of Buffalo, class of 1872.

Dr. Cochrane was born in Lyndonville in 1843. He is survived by his widow, two sons, Dr. William Cochrane, of Rochester, and Dr. Samuel Cochrane, of Batavia, and a sister, Mrs. Mary Babcock. The remains were taken to Albion for interment.

